# Psychometric validation of the Chinese Warwick–Edinburgh mental well-being scale in patients with heart failure or myocardial infarction: evidence from classical test theory and item response theory

**DOI:** 10.3389/fpsyt.2026.1797338

**Published:** 2026-07-15

**Authors:** Qinyi Tang, Xinrui Zhang, Haitao Zhou, Rulian Zheng, Shaoting Hu, Wenjian Guo, Aishu Dong

**Affiliations:** The Second Affiliated Hospital of Wenzhou Medical University, Wenzhou, Zhejiang, China

**Keywords:** cardiovascular care, Chinese Warwick-Edinburgh mental well-being scale, classical test theory, heart failure, item response theory, myocardial infarction, positive mental well-being, psychometric validation

## Abstract

**Background:**

Positive mental well-being is increasingly recognized as an important component of cardiovascular care, particularly among patients with heart failure or myocardial infarction. However, validated Chinese-language instruments for assessing positive mental well-being in this population remain limited.

**Objective:**

This study evaluated the psychometric properties of the Chinese-language 14-item Warwick–Edinburgh Mental Well-Being Scale (WEMWBS) in patients with heart failure or myocardial infarction.

**Methods:**

In this cross-sectional validation study, 392 patients with heart failure or myocardial infarction completed the Chinese-language WEMWBS. Construct validity was examined using exploratory and confirmatory factor analyses. Convergent validity was assessed using factor loadings, composite reliability (CR), and average variance extracted (AVE), and criterion-related validity was evaluated using the 5-item World Health Organization Well-Being Index (WHO-5) as an external criterion. Internal consistency was estimated using Cronbach’s α. Item-level performance was examined using item response theory under the graded response model.

**Results:**

The Chinese-language WEMWBS showed excellent internal consistency, with Cronbach’s α = 0.961. The modified one-factor confirmatory factor model showed acceptable fit on most indices, with χ²/df = 2.68, CFI = 0.955, TLI = 0.944, RMSEA = 0.091, and SRMR = 0.038. Criterion-related validity was supported by a strong positive correlation with the WHO-5 (r = 0.746). Item response theory analyses indicated generally satisfactory item discrimination and ordered threshold parameters.

**Conclusions:**

The Chinese-language WEMWBS demonstrated satisfactory psychometric properties in patients with heart failure or myocardial infarction. These findings support its potential use as a patient-reported outcome measure for assessing positive mental well-being in this population. Longitudinal and intervention studies are needed to evaluate its responsiveness, predictive validity, and clinical utility in cardiovascular care.

## 1 Introduction

Mental well-being is an important component of cardiovascular care, particularly for patients with heart failure or myocardial infarction. Cardiovascular diseases (CVDs) impose a substantial health burden worldwide and remain a major public health challenge in China. In 2022, CVD accounted for 48.00% of deaths in rural areas and 45.86% of deaths in urban areas in China. Approximately 330 million people in China are affected by CVD, including 11.39 million with coronary heart disease and 8.9 million with heart failure (1–3). These figures underscore the clinical and public health importance of addressing both physical and psychological health in cardiovascular populations.

Despite advances in cardiovascular treatment, patients with heart failure or myocardial infarction often experience persistent symptoms, functional limitations, repeated healthcare use, and psychological burden (4). Anxiety, depressive symptoms, social isolation, and reduced well-being are common in this population (5). Poor mental well-being may adversely affect treatment adherence, rehabilitation engagement, self-management, physical activity, quality of life, and prognosis. Therefore, valid assessment of mental well-being is clinically relevant for patients with heart failure or myocardial infarction.

Positive psychology offers a complementary framework for assessing mental health in cardiovascular populations (6). Rather than focusing solely on psychopathology, it emphasizes strengths, positive emotions, psychological functioning, and well-being (7). From this perspective, mental health reflects not only the absence of disorder but also positive psychological attributes and functioning (8), including both hedonic and eudaimonic dimensions (9). The WEMWBS was developed within this positive mental health framework and assesses positive well-being rather than psychological distress alone. It may therefore complement symptom-focused measures and provide a broader assessment of psychological health in patients with heart failure or myocardial infarction (10).

In chronic disease care, psychological stress may be shaped not only by the disease itself but also by reduced social support, medical costs, functional limitations, and uncertainty about prognosis (11). These factors can deplete psychological resources and make it more difficult for patients to maintain positive mental health. Integrating positive psychology into cardiovascular care may therefore support a broader model of care that addresses distress while also promoting resilience, well-being, and quality of life in vulnerable populations.

In clinical practice, instruments such as the Hospital Anxiety and Depression Scale, the Patient Health Questionnaire-9, and the Generalized Anxiety Disorder-7 are commonly used to screen for anxiety and depressive symptoms. Although these tools are valuable for identifying psychological distress, they focus primarily on negative symptoms and may not capture positive dimensions of mental health. The WEMWBS is a brief, acceptable, and easy-to-administer measure of positive mental well-being that covers positive affect, psychological functioning, and interpersonal aspects of well-being. Accordingly, it should be viewed as a complement to, rather than a replacement for, symptom-focused measures in cardiovascular care.

For patients with heart failure or myocardial infarction, the WEMWBS may have potential value in cardiac rehabilitation, nursing follow-up, psychological support, and patient-reported outcome assessment. However, these potential applications depend on adequate psychometric performance in the intended clinical population. Therefore, the Chinese-language WEMWBS requires evaluation among patients with heart failure or myocardial infarction before it can be used confidently in cardiovascular care and research.

The WEMWBS (12), developed by Tennant et al. in 2007, is a well-established instrument for evaluating mental well-being. It captures multiple aspects of positive mental health, including positive affect, positive psychological functioning, and fulfilling interpersonal relationships. Extensive research has supported the reliability, validity, and cultural adaptability of the WEMWBS across diverse populations (13–16). The scale has also been translated into numerous languages, several of which have demonstrated good psychometric properties (17–20). Nevertheless, further evidence is needed in clinical populations, particularly among patients with cardiovascular conditions such as heart failure or myocardial infarction.

Classical test theory (CTT) has traditionally been used to evaluate instruments such as the WEMWBS, providing evidence on reliability, item–total correlations, factor structure, and associations with external measures (13, 20). However, CTT provides limited information about how individual items perform across different levels of the latent trait. Item response theory (IRT) offers complementary item-level information, including item discrimination, threshold parameters, item characteristic curves, item information curves, test information functions, and measurement precision across the well-being continuum (21, 22). Combining CTT and IRT may therefore provide a more comprehensive evaluation of the Chinese-language WEMWBS.

Previous studies have examined the Chinese-language WEMWBS in several populations, including older adults, university students, medical staff, and patients with chronic heart failure. However, important gaps remain. First, psychometric evidence remains limited in cardiovascular populations, particularly among patients with myocardial infarction or mixed cardiovascular conditions. Patients with heart failure or myocardial infarction may experience persistent symptoms, functional limitations, repeated healthcare use, prognostic uncertainty, and psychological burden, all of which may influence their responses to well-being items. Therefore, findings from other populations may not be fully generalizable to this clinical group. Second, previous Chinese-language studies have focused mainly on scale-level reliability and validity, whereas item-level performance in cardiovascular patients remains insufficiently examined. Third, although IRT has been used to evaluate the WEMWBS in some populations, evidence regarding item discrimination, response threshold parameters, item information, and measurement precision in patients with heart failure or myocardial infarction remains limited.

This study evaluated the psychometric properties of the Chinese-language WEMWBS in patients with heart failure or myocardial infarction. Using both CTT and IRT, we examined scale-level reliability, structural validity, convergent validity, criterion-related validity, differential item functioning, item discrimination, threshold parameters, item information, and measurement precision. The study aimed to provide psychometric evidence for the potential use of the Chinese-language WEMWBS as a patient-reported measure of positive mental well-being in cardiovascular care.

## 2 Methods

### 2.1 Reporting guideline

This study was reported in accordance with the Strengthening the Reporting of Observational Studies in Epidemiology (STROBE) guideline for cross-sectional studies.

### 2.2 Participants and data collection

This study used a cross-sectional design, with all data collected at a single time point at patient enrollment; no follow-up assessments were conducted. A cross-sectional design was appropriate for the initial psychometric validation of the Chinese-language WEMWBS in this clinical population because the primary aim was to examine its factor structure, reliability, convergent validity, and item-level performance.

Between January and October 2023, 392 patients diagnosed with heart failure or myocardial infarction were recruited from tertiary hospitals in mainland China. A purposive sampling strategy was used to select tertiary hospital settings, within which eligible patients were approached consecutively. Trained researchers screened newly admitted patients daily during the study period. Patients who met the predefined inclusion criteria were informed about the study purpose and invited to participate until the target sample size was achieved. Before enrollment, all participants received written information detailing the study aims, procedures, and ethical considerations.

Data were collected using paper-based questionnaires, which were administered and retrieved on-site by trained researchers to minimize missing data and improve data quality. Participants were informed that participation was voluntary and that all personal information and responses would be kept confidential, de-identified, and used only for research purposes.

### 2.3 Sample size calculation

We estimated the sample size *a priori* using G*Power v3.1.9.7. For convergent validity, the correlation: bivariate normal model (two-tailed), effect size r = 0.30, α = 0.05, and power (1–β) = 0.80 yielded N = 84. For internal consistency, assuming Cronbach’s α = 0.85 with a 95% CI half-width ≤ 0.05 (Bonett precision), the recommended sample size was N ≥ 150. For factor analysis, we used the criterion of ≥10 participants per item and a minimum of N ≥ 200 for stable CFA estimation. The actual enrollment was 392, which satisfied all *a priori* requirements for reliability and validity testing.

### 2.4 Inclusion criteria

Eligible participants were patients with a definitive diagnosis of heart failure or myocardial infarction who were in a stable clinical condition, were aged 18 years or older, were conscious, and were able to cooperate with the research procedures.

### 2.5 Exclusion criteria

Patients were excluded if they had: (1) severe cognitive impairment; (2) a diagnosed severe psychiatric disorder; (3) severe acute illness preventing questionnaire completion; or (4) incomplete questionnaire data.

Participants with diagnosed severe psychiatric disorders were excluded to minimize potential confounding in scale validation, because such conditions may substantially alter the perception and interpretation of well-being items. This exclusion did not apply to patients with mild-to-moderate depressive or anxiety symptoms, which are common among individuals with heart failure or myocardial infarction.

### 2.6 Instruments

The WEMWBS (12) is a 14-item instrument that uses a 5-point Likert scale ranging from “none of the time” to “all of the time,” with each item scored from 1 to 5. Total scores range from 14 to 70, with higher scores indicating greater positive mental well-being. The scale was designed for population-level surveys and provides a concise measure of overall mental well-being. In this study, the Chinese-language version of the WEMWBS was used, which has shown acceptable reliability and validity in previous Chinese populations. The WEMWBS may help researchers and clinicians assess positive mental well-being and identify individuals who may benefit from psychosocial support (23).

The 5-item World Health Organization Well-Being Index (WHO-5) is a widely used tool for assessing subjective psychological well-being (24). Developed by the World Health Organization, the scale comprises five positively worded items assessing positive mood, vitality, and general interest, with higher scores indicating better well-being (25). The WHO-5 has been widely used in clinical and epidemiological studies and in evaluations of psychological well-being interventions (26–29). Because it assesses subjective well-being and is brief enough for clinical use, the WHO-5 was selected as the comparator measure for examining the criterion-related validity of the Chinese-language WEMWBS (12).

### 2.7 Statistical analysis

Psychometric analyses were conducted using both CTT (30) and IRT (21) to evaluate the performance of the WEMWBS in a cardiovascular clinical population. Analyses were performed using AMOS version 26.0, SPSS version 27.0, and R version 4.3.3.

Descriptive statistics, including means and standard deviations (SDs) for continuous variables and frequencies and percentages for categorical variables, were calculated to summarize sample characteristics. A two-sided P value < 0.05 was considered statistically significant where applicable.

#### 2.7.1 Preliminary dimensionality assessment

To examine the preliminary dimensionality of the Chinese-language WEMWBS, exploratory factor analysis (EFA) using principal axis factoring (PAF) was conducted on the 14 items (31). The proportion of variance explained by the first factor was examined to assess whether a dominant general factor was present. This analysis provided empirical support for the subsequent confirmatory factor analysis.

#### 2.7.2 Floor and ceiling effects

Floor and ceiling effects were examined to assess the sensitivity of the WEMWBS in measuring variation in mental well-being. Descriptive statistics, including the frequency of responses at the lowest and highest scale scores, were calculated using SPSS. A floor or ceiling effect was considered present if more than 15% of participants scored at the minimum or maximum, respectively (32).

#### 2.7.3 Item analysis

Item discrimination refers to the ability of an item to distinguish between respondents with different levels of the underlying trait. Independent-samples t-tests were used to compare the mean item scores between the high-score group (top 27% of respondents) and the low-score group (bottom 27% of respondents) based on their total scale scores (33). Items that failed to show adequate discrimination were considered for revision or removal in conjunction with other psychometric evidence.

#### 2.7.4 Reliability of the WEMWBS

Internal consistency was evaluated using Cronbach’s α, with values ≥ 0.70 considered acceptable. To further examine internal consistency, split-half reliability was assessed using both sequential and odd-even split methods. A correlation coefficient (r) ≥ 0.70 was considered acceptable. This approach estimates consistency between two equivalent halves of the scale.

#### 2.7.5 Validity of the WEMWBS

##### 2.7.5.1 Convergent validity

Convergent validity was evaluated to determine whether items intended to measure the same latent construct were sufficiently interrelated. This analysis focused on internal coherence among items within the construct rather than on associations with external criteria. Factor loadings obtained from CFA were used to compute AVE and CR for the construct. An AVE ≥ 0.50 and a CR ≥ 0.70 were considered indicative of acceptable convergent validity.

##### 2.7.5.1 Construct validity

###### 2.7.5.1.1 Sample splitting strategy

To examine the factor structure of the Chinese-language WEMWBS, the total sample (N = 392) was randomly split into two independent subsamples: one for EFA (n = 190) and one for CFA (n = 202). The CFA subsample included more than 200 participants to support stable parameter estimation, while the EFA subsample exceeded the commonly recommended minimum of 5–10 participants per item. Demographic characteristics were compared between the two subsamples to assess whether the random split introduced systematic differences.

###### 2.7.5.1.2 Construct validity: exploratory factor analysis

EFA was conducted on the EFA subsample using PAF. Sampling adequacy and suitability for factor analysis were evaluated using the Kaiser–Meyer–Olkin (KMO) measure and Bartlett’s test of sphericity. The number of factors to retain was determined using parallel analysis. Factor structure was evaluated using factor loadings, communalities, variance explained, and model fit indices, including RMSR, TLI, RMSEA, and factor score correlation.

###### 2.7.5.1.3 Construct validity: confirmatory factor analysis

CFA was performed on the CFA subsample (n = 202) to validate the unidimensional structure suggested by EFA (34). The MLM estimator was used. Model fit was evaluated using the comparative fit index (CFI > 0.95), Tucker–Lewis index (TLI > 0.95), root mean square error of approximation (RMSEA < 0.06), and standardized root mean square residual (SRMR < 0.08). If the initial model showed suboptimal fit, residual correlations were allowed only when supported by both modification indices and theoretical justification, while retaining the unidimensional factor structure.

##### 2.7.5.2 Criterion-related validity

Criterion-related validity was assessed by examining the association between WEMWBS scores and an external, established measure of well-being. Pearson correlation coefficients were calculated between WEMWBS scores and WHO-5 scores. The WHO-5 was selected as the external criterion because of its brevity, international recognition, and well-established psychometric properties in general and clinical populations. The WHO-5 assesses positive dimensions of well-being, including emotional state, energy, positive thinking, daily interest, and life satisfaction, which are conceptually aligned with the construct measured by the WEMWBS. Correlation coefficients greater than 0.50 were interpreted as strong, coefficients from 0.30 to 0.50 as moderate, and coefficients below 0.30 as weak.

#### 2.7.6 Differential item functioning

Differential item functioning (DIF) across gender was examined using the lordif package in R under the graded response model (GRM). Participants were divided into males (reference group, n = 264) and females (focal group, n = 128). An iterative purification procedure (maximum 10 iterations) was applied to obtain unbiased trait estimates. DIF was tested using ordinal logistic regression to examine both uniform and non-uniform DIF. A two-step decision rule was applied: statistical significance at α = 0.01 using the likelihood ratio chi-square test and effect size measured by the change in Nagelkerke pseudo-R² (ΔR²). Following Zumbo, Jodoin, and Gierl, items with ΔR² ≥ 0.02 and p < 0.01 were flagged as showing practically meaningful DIF. Items meeting only the significance criterion were interpreted as negligible. Monte Carlo simulations with 500 replications were conducted to enhance robustness (35, 36).

#### 2.7.7 Item response theory analysis

The psychometric properties of the Chinese-language WEMWBS were analyzed using IRT. Before fitting the IRT model, the assumptions of unidimensionality and local independence were evaluated. Local independence was assessed by examining residual correlations among items after accounting for the latent trait, with residual correlations < 0.10 considered acceptable. Unidimensionality was examined using CFA to assess whether item correlations could be adequately explained by a single latent factor. Evidence supporting unidimensionality and local independence was considered necessary for IRT analysis.

Given the ordered polytomous response format of the Chinese-language WEMWBS, which uses a 5-point Likert scale, the GRM (37) was adopted for item analysis. To empirically justify this choice, three unidimensional IRT models were compared: the freely estimated GRM, the freely estimated generalized partial credit model (GPCM), and a constrained GRM with all item discrimination parameters fixed to be equal. Model fit was evaluated using the Akaike information criterion (AIC) and Bayesian information criterion (BIC), and nested models were compared using likelihood ratio tests. As shown in **Table 1**, the freely estimated GRM yielded substantially lower AIC (11158.46) and BIC (11436.45) than the GPCM (ΔAIC = 92, ΔBIC = 92), and the equal-discrimination GRM fit significantly worse than the free GRM (p <.001), indicating non-negligible variation in discrimination parameters across items. Category thresholds were ordered for all 14 items, supporting the appropriateness of the cumulative probability structure assumed by the GRM. Therefore, the GRM was retained as the final measurement model (**Table 1**).

**Table 1:** Model fit indices for competing IRT models.

Model	N parameters	AIC	BIC	LRT p (vs. free GRM)
GRM (free a)	70	11158.46	11436.45	—
GPCM (free a)	70	11250.50	11528.48	—
GRM (equal a)	57	11384.81	11611.17	<0.001

GRM, graded response model; GPCM, generalized partial credit model; AIC, Akaike information criterion; BIC, Bayesian information criterion; LRT, likelihood ratio test. Free a, item discrimination parameters freely estimated; equal a, all item discrimination parameters constrained to be equal.

Item information curves (IICs) were examined to evaluate the amount of information provided by each item across the latent trait continuum, and item characteristic curves (ICCs) were examined to evaluate response category functioning. The test information function (TIF) was used to assess measurement precision at the scale level across the latent trait continuum. Ideally, category response curves should show ordered, distinct peaks as the latent trait increases. Higher item information values indicate lower measurement standard errors. The shape of each IIC was inspected to identify the range of the latent trait over which the item provided the most information. Ideally, the TIF should provide relatively high information across a broad range of the latent trait, indicating stable measurement precision across that range (38).

## 3 Results

### 3.1 Participant characteristics and descriptive statistics

**Figure 1:**
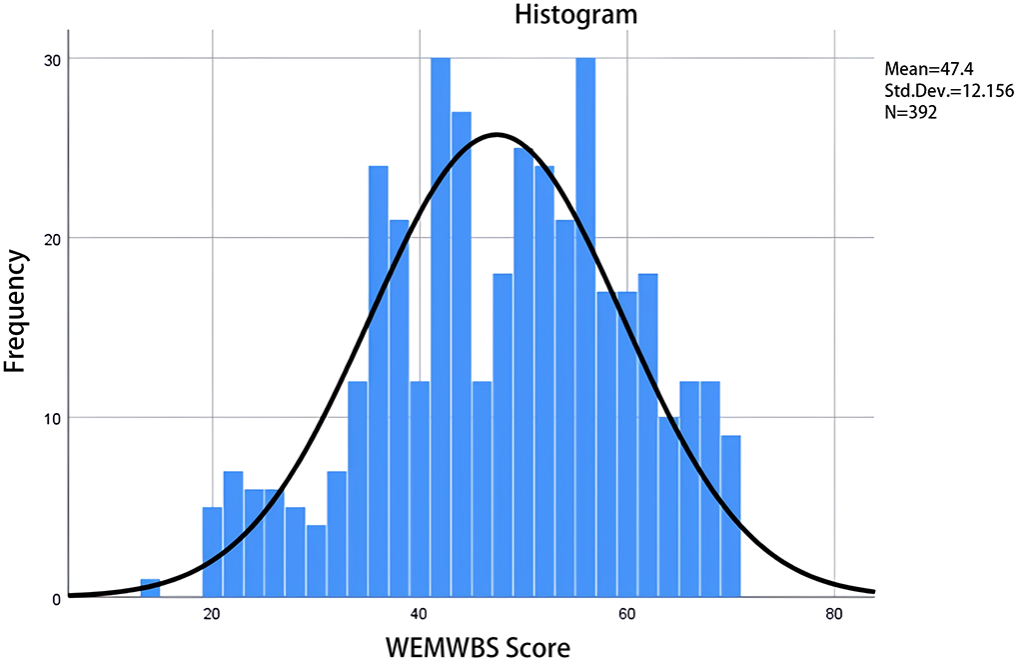
Histogram of Chinese-language WEMWBS scores among participants (N = 392).

Descriptive statistics were computed for WEMWBS scores across the entire cohort (**Figure 1**). The mean score was 47.4 (SD = 12.156), indicating moderate positive mental well-being on average. The score distribution was approximately normal, with a slight negative skew toward higher levels of well-being. As shown in **Table 2**, the skewness coefficient was –0.264 and the kurtosis coefficient was –0.518.

**Table 2:** Skewness and kurtosis values for Chinese-language WEMWBS scores.

Valid N	Missing N	Skewness	Std. error of skewness	Kurtosis	Std. error of kurtosis
392	0	–0.264	0.123	–0.518	0.246

The study included 392 participants with a mean age of 70.94 (SD = 11.95) years. Of these, 67.3% were male and 32.7% were female. Most participants were married (85.2%), and 62.5% had primary education or below. Regarding lifestyle factors, 54.1% were non-drinkers, 43.1% were non-smokers, and 37.8% smoked fewer than 10 cigarettes per day (**Table 3**).

**Table 3:** Demographic characteristics and comparability of the EFA and CFA subsamples (N = 392).

Variable	Category	Total (N = 392)	EFA (n = 190)	CFA (n = 202)	Test statistic
Gender, n (%)	Male	264 (67.3)	135 (71.1)	129 (63.9)	χ²(1) = 0.92, p = 0.337
	Female	128 (32.7)	55 (28.9)	73 (36.1)	
Age (years), M (SD)		70.94 (11.95)	69.98 (11.90)	71.83 (11.97)	t(387.29) = 1.53, p =0.127
Education, n (%)	Primary or below	245 (62.5)	114 (60.0)	131 (64.9)	χ²(4) = 1.54 p = 0.819
	Junior high	90 (23.0)	47 (24.7)	43 (21.3)	
	Senior high	28 (7.1)	13 (6.8)	15 (7.4)	
	College	16 (4.1)	8 (4.2)	8 (4.0)	
	Postgraduate	13 (3.3)	8 (4.2)	5 (2.5)	
Marital status, n (%)	Married	334 (85.2)	157 (82.6)	177 (87.6)	χ²(3) = 2.76 p =0.430
	Unmarried	7 (1.8)	3 (1.6)	4 (2.0)	
	Divorced	7 (1.8)	5 (2.6)	2 (1.0)	
	Widowed	44 (11.2)	25 (13.2)	19 (9.4)	
Smoking, n (%)	Never	169 (43.1)	83 (43.7)	86 (42.6)	χ²(4) = 4.81, p =0.308
	Occasionally	63 (16.1)	30 (15.8)	33 (16.3)	
	Sometimes	148 (37.8)	67 (35.3)	81 (40.1)	
	Often	8 (2.0)	7 (3.7)	1 (0.5)	
	Daily	4 (1.0)	3 (1.6)	1 (0.5)	
Alcohol, n (%)	Never	212 (54.1)	105 (55.3)	107 (53.0)	χ²(3) = 1.38, p = 0.710^a^
	Occasionally	70 (17.9)	36 (18.9)	34 (16.8)	
	Sometimes	87 (22.2)	39 (20.5)	48 (23.8)	
	Often	23 (5.9)	10 (5.3)	13 (6.4)	
	Daily	0 (0.0)	0 (0.0)	0 (0.0)	

EFA, exploratory factor analysis group; CFA, confirmatory factor analysis group. Continuous variables are presented as M (SD); categorical variables as n (%). Group differences were examined using an independent-samples t-test for age and chi-square tests for categorical variables.

^a^ Due to zero frequency in the “Daily” category, alcohol use was collapsed into four categories for the chi-square test.

### 3.2 Item analysis

Respondents were classified into high- and low-score groups based on their total scale scores, and item discrimination was analyzed using independent-samples t-tests. As summarized in **Table 4**, significant differences were observed between the two groups for all 14 items (P < 0.001), indicating that the items distinguished between respondents with higher and lower levels of positive mental well-being. Therefore, no items were removed. Item–total correlation coefficients ranged from r = 0.604 to r = 0.896, indicating strong associations between individual items and total scale scores.

**Table 4:** Item discrimination and item–scale correlations for the Chinese-language WEMWBS (N = 392).

Item	Correlations with total score	Discrimination between items		
	Item-scale correlation, r	High-score group (N = 109), mean (SD)	Low-score group (N = 110), mean (SD)	t score
1	0.867***	4.61 (0.56)	2.13 (0.78)	–27.147***
2	0.882***	4.37 (0.62)	1.95 (0.70)	–27.122***
3	0.800***	4.03 (0.73)	2.42 (0.67)	–22.674***
4	0.795***	4.43 (0.57)	2.02 (0.78)	–19.757***
5	0.823***	4.46 (0.54)	1.63 (0.57)	–25.965***
6	0.866***	4.45 (0.57)	2.19 (0.74)	–25.252***
7	0.819***	4.72 (0.45)	2.40 (0.78)	–22.676***
8	0.894***	4.54 (0.57)	2.09 (0.67)	–28.856***
9	0.705***	4.61 (0.56)	3.18 (0.74)	–17.555***
10	0.896***	4.37 (0.63)	1.97 (0.72)	–28.193***
11	0.806***	4.03 (0.73)	2.55 (0.82)	–21.239***
12	0.604***	4.43 (0.57)	3.48 (0.91)	–12.762***
13	0.782***	4.46 (0.54)	1.89 (0.78)	–19.660***
14	0.834***	4.45 (0.57)	2.36 (0.83)	–22.616***

**P < 0.01, ***P< 0.001.

As shown in **Table 5**, no item exceeded the 15% threshold for floor effects. However, several items showed ceiling effects greater than 15%, suggesting that a substantial proportion of respondents selected the highest response category for those items.

**Table 5:** Floor and ceiling effects for the Chinese-language WEMWBS. Floor and ceiling effects were defined as endorsement of the lowest (1 point) and highest (5 points) response categories, respectively (N = 392).

Item	M	SD	Subjects with floor effect, N (%)	Subjects with a ceiling effect, N (%)
1	3.38	1.169	16 (0.04)	77 (0.20)
2	3.21	1.128	21 (0.05)	53 (0.14)
3	3.45	1.033	8 (0.02)	71 (0.18)
4	2.41	1.266	15 (0.04)	35 (0.09)
5	2.76	1.149	42 (0.11)	30 (0.08)
6	3.32	1.079	14 (0.04)	57 (0.15)
7	3.44	1.025	9 (0.02)	59 (0.15)
8	3.33	1.097	14 (0.04)	58 (0.15)
9	4.03	0.847	1 (0.00)	122 (0.31)
10	3.32	1.460	22 (0.06)	60 (0.15)
11	3.56	1.044	7 (0.02)	82 (0.21)
12	4.18	0.807	2 (0.01)	143 (0.36)
13	2.40	1.274	31 (0.08)	31 (0.08)
14	3.48	1.061	12 (0.03)	70 (0.18)

### 3.3 Reliability analysis

The scale demonstrated excellent internal consistency (Cronbach’s α = 0.961). Split-half reliability yielded a coefficient of 0.961, indicating strong consistency between the two halves of the scale. All corrected item–total correlation coefficients were greater than 0.50 (**Table 6**), indicating adequate associations between individual items and the total scale score.

**Table 6:** Cronbach’s reliability analysis of the Chinese-language WEMWBS (N = 392).

Item	Average score after deleting each item	Scaled variance after deleting terms	Corrected item-total correlation	Squared multiple correlation	Cronbach’s alpha if the item is deleted
1	44.02	124.506	0.839	0.778	0.957
2	44.19	124.873	0.858	0.790	0.956
3	43.95	128.765	0.766	0.642	0.959
4	44.29	128.159	0.759	0.650	0.959
5	44.64	126.107	0.788	0.662	0.958
6	44.08	126.221	0.841	0.816	0.957
7	43.96	128.431	0.788	0.766	0.958
8	44.07	125.136	0.873	0.783	0.956
9	43.41	133.639	0.666	0.579	0.961
10	44.13	123.953	0.875	0.798	0.956
11	43.83	128.389	0.773	0.739	0.958
12	43.28	136.101	0.557	0.460	0.962
13	44.42	127.523	0.741	0.633	0.959
14	43.91	127.389	0.804	0.715	0.958

### 3.4 Construct validity: exploratory factor analysis

To explore the underlying factor structure of the Chinese-language WEMWBS in this population, EFA was first conducted on a random subsample.

The KMO measure of sampling adequacy was 0.96, and Bartlett’s test of sphericity was significant, χ²(91) = 2521.77, p <.001, indicating that the data were highly suitable for factor analysis. Parallel analysis suggested retention of one factor (**Table 7**). When a single-factor solution was forced, factor loadings ranged from 0.61 to 0.88, and the single factor accounted for 64% of the total variance. The regression factor score correlation was 0.98, and the minimum possible correlation was 0.93, indicating excellent reliability and stability of the factor scores (**Table 8**).

**Table 7:** Factor loadings, communalities, and fit indices from exploratory factor analysis (PAF) for the Chinese-language WEMWBS (n = 190).

Item	Factor loading	h²	u²
1	0.85	0.72	0.28
2	0.88	0.78	0.22
3	0.78	0.60	0.40
4	0.79	0.63	0.37
5	0.82	0.68	0.32
6	0.85	0.72	0.28
7	0.80	0.65	0.35
8	0.88	0.78	0.22
9	0.69	0.47	0.53
10	0.88	0.77	0.23
11	0.80	0.64	0.36
12	0.61	0.37	0.63
13	0.73	0.54	0.46
14	0.80	0.64	0.36
Summary statistics			
Eigenvalue		8.97	
Variance explained		64%	
KMO		0.96	
Bartlett’s test of sphericity		χ²(91) = 2521.77, p <.001	

PAF, principal axis factoring; h², communality; u², uniqueness. All factor loadings were significant at p <.001. The KMO and Bartlett’s tests confirmed the adequacy of the data for factor analysis.

**Table 8:** Model fit indices from the EFA model (n = 190).

Fit index	Value	Criterion
RMSR	0.06	< 0.08
TLI	0.851	> 0.80
RMSEA	0.145 [90% CI: 0.131, 0.160]	—
Factor score correlation	0.98	—
Minimum possible correlation	0.93	> 0.50

RMSR, root mean square residual; TLI, Tucker–Lewis index; RMSEA, root mean square error of approximation; CI, confidence interval. RMSEA is typically higher in EFA because of the absence of a fixed model structure and is less central for interpretation at this stage.

All 14 items demonstrated adequate loadings (> 0.60), supporting a unidimensional structure in this sample.

### 3.5 Construct validity: confirmatory factor analysis

To confirm the unidimensional structure suggested by EFA, CFA was performed on the second independent subsample. Using the CFA subsample (n = 202), a one-factor confirmatory factor model was fitted to the 14 items.

The initial model showed suboptimal fit (χ²/df = 5.04, CFI = 0.886, TLI = 0.865, RMSEA = 0.141, SRMR = 0.055). Based on modification indices and theoretical considerations, four pairs of residual correlations were allowed to covary: item 7–item 11 (MI = 65.43), item 6–item 7 (MI = 60.16), item 6–item 11 (MI = 38.97), and item 9–item 12 (MI = 31.12). These pairs reflected conceptually related content: item 7 (“I’ve been thinking clearly”) and item 11 (“I’ve been able to make decisions”) both reflect cognitive functioning; item 6 (“I’ve been feeling optimistic”) and item 7 both pertain to positive cognitive outlook; item 6 and item 11 are linked through positive cognitive appraisal; and item 9 (“I’ve been feeling close to other people”) and item 12 (“I’ve been feeling loved”) both tap interpersonal connectedness and social relationships.

The modified model showed improved fit (χ²(73) = 195.37, χ²/df = 2.68, CFI = 0.955, TLI = 0.944, RMSEA = 0.091, 90% CI [0.076, 0.107], SRMR = 0.038). All standardized factor loadings were significant (p <.001) and ranged from 0.536 to 0.909 (**Table 9**).

**Table 9:** Model fit indices and standardized factor loadings for the one-factor CFA model (n = 202).

Panel A. Fit indices										
Model	χ²		df	χ²/df	CFI		TLI	RMSEA (90% CI)		SRMR
Initial model	388.24		77	5.04	0.886		0.865	0.141 (0.128, 0.156)		0.055
Modified model	195.37		73	2.68	0.955		0.944	0.091 (0.076, 0.107)		0.038
Panel B. Standardized factor loadings (modified model)										
Item		Sth. loading				Item			Sth. loading	
1		0.881				8			0.904	
2		0.882				9			0.659	
3		0.797				10			0.909	
4		0.759				11			0.744	
5		0.792				12			0.536	
6		0.841				13			0.776	
7		0.768				14			0.845	

CFA, confirmatory factor analysis; CFI, comparative fit index; TLI, Tucker–Lewis index; RMSEA, root mean square error of approximation; SRMR, standardized root mean square residual; CI, confidence interval. The modified model included four correlated residuals: item7–item11, item6–item7, item6–item11, and item9–item12. All factor loadings were significant at p <.001.

### 3.6 Convergent validity

After structural validity was examined, convergent validity was assessed using factor loadings, CR, and AVE. CR and AVE were calculated in R. As shown in **Table 10**, factor loadings ranged from 0.546 to 0.902. The CR value exceeded 0.70, and the AVE value exceeded 0.50, supporting acceptable convergent validity for the unidimensional construct.

**Table 10:** Convergent validity based on factor loadings, CR, and AVE (N = 392).

Item	Estimate	S.E.	CR	AVE
1	0.864		0.963**	0.66**
2	0.891	0.040		
3	0.785	0.041		
4	0.766	0.043		
5	0.814	0.044		
6	0.832	0.041		
7	0.769	0.041		
8	0.902	0.038		
9	0.657	0.038		
10	0.900	0.040		
11	0.754	0.042		
12	0.546	0.039		
13	0.748	0.046		
14	0.807	0.035		

**P < 0.01

Given the unidimensional structure of the WEMWBS, as confirmed by CFA, discriminant validity analysis was not applicable and was therefore not performed.

### 3.7 Criterion-related validity

Criterion-related validity was assessed by examining the correlation between the Chinese-language WEMWBS and the WHO-5. A significant positive correlation was observed (r = 0.746; 95% CI: 0.722–0.794; P < 0.01), indicating a strong association between the two measures and supporting the criterion-related validity of the Chinese-language WEMWBS in this population.

### 3.8 Differential item functioning

Gender-related DIF was examined in R using the lordif package (version 1.0-1) under the GRM. The ΔR² value was used as the effect size measure, with a threshold of 0.02 for practically meaningful DIF (35, 36).

As shown in **Table 11**, the chi-square tests indicated statistical significance for items 3, 4, 6, 9, 11, and 14 (p < 0.01). However, ΔR² values for all 14 items ranged from 0.0000 to 0.0163, all below the 0.02 threshold. According to the effect size criterion, no item showed practically meaningful DIF. These results provide no evidence of practically meaningful gender-related DIF in the present sample.

**Table 11:** Differential item functioning analysis across gender groups.

Item	χ² (p12)	p-value	ΔR² (Nagelkerke)	DIF status
1	0.3862	0.3389	0.0005	No DIF
2	0.6222	0.4301	0.0001	No DIF
3	0.0015	0.9687	0.0107	No DIF
4	0.0041	0.9486	0.0163	No DIF
5	0.1457	0.7026	0.0020	No DIF
6	0.0005	0.9820	0.0071	No DIF
7	0.0157	0.9000	0.0050	No DIF
8	0.1533	0.6953	0.0010	No DIF
9	0.0019	0.9655	0.0162	No DIF
10	0.8552	0.3550	0.0000	No DIF
11	0.0000	0.9999	0.0160	No DIF
12	0.0194	0.8891	0.0123	No DIF
13	0.5028	0.4783	0.0008	No DIF
14	0.0041	0.9486	0.0070	No DIF

χ² (p12), chi-square statistic for uniform DIF; ΔR², change in Nagelkerke pseudo-R². An item was flagged as showing practically meaningful DIF if ΔR² ≥ 0.02. All items showed ΔR² < 0.02, indicating no practically meaningful DIF.

### 3.9 Item response theory analysis

#### 3.9.1 Item discrimination and thresholds

The R analyses yielded item discrimination parameters (a), threshold parameters (b), ICCs, and IICs for each WEMWBS item. As shown in **Table 12**, the threshold parameters ranged from –2.61 to 2.77, suggesting that the items covered a broad range of the latent well-being continuum.

**Table 12:** Item discrimination parameters (a) and threshold parameters (b) for the Chinese-language WEMWBS (N = 392).

Item	a	b1	b2	b3	b4
1	2.55	−1.53	−0.63	0.47	1.94
2	2.29	−2.01	−0.68	0.43	2.02
3	1.76	−2.61	−0.63	0.74	2.77
4	1.29	−2.57	−0.89	0.39	2.58
5	1.69	−1.45	−0.13	1.15	2.54
6	2.28	−2.01	−0.69	0.61	1.98
7	2.17	−2.25	−1.09	0.17	1.73
8	3.83	−1.44	−0.45	0.52	1.58
9	1.87	−1.73	−0.52	0.51	1.97
10	3.28	−1.31	−0.38	0.54	1.60
11	1.68	−2.35	−1.26	0.07	1.61
12	1.69	−2.05	−0.82	0.20	1.41
13	1.83	−1.98	−0.87	0.34	1.72
14	3.56	−1.54	−0.55	0.47	1.81

Item discrimination parameters (a) ranged from 1.29 to 3.83. Item 8 (WEMWBS8; a = 3.83) showed the highest discrimination, followed by item 14 (WEMWBS14; a = 3.56) and item 10 (WEMWBS10; a = 3.28). Item 4 (WEMWBS4; a = 1.29) showed the lowest discrimination. These results indicate that most items had moderate to high discriminatory power.

Threshold parameters increased progressively across all items from the first to the last threshold, indicating ordered response categories. Higher item scores were associated with higher levels of self-reported psychological well-being, although the rate of increase varied across items.

#### 3.9.2 Item characteristic curves

**Figure 2:**
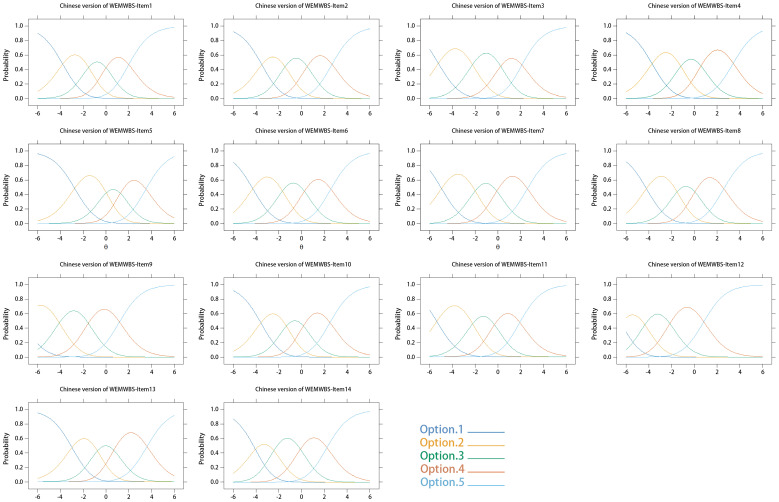
Item characteristic curves for the Chinese-language WEMWBS. Each curve represents the probability of endorsing a response category for each item (N = 392).

The ICCs for each item are shown in **Figure 2**. These curves illustrate how the probability of endorsing each WEMWBS response category (from “none of the time” to “all of the time”) varies across levels of the latent trait. The curves are influenced by both threshold parameters and discrimination parameters. Items with higher discrimination values show steeper changes in response probability across levels of the latent trait (39). In this study, most items showed ordered category response patterns, supporting the response structure assumed by the GRM.

#### 3.9.3 Item information and expected score curves

**Figure 3:**
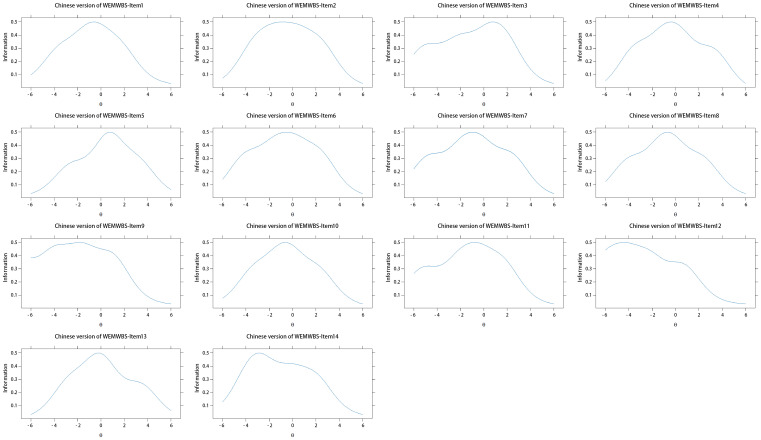
Item information curves for the Chinese-language WEMWBS. Each curve represents the amount of information provided by an item across the latent trait continuum (N = 392).

The IICs for the WEMWBS are shown in **Figure 3**. The curves indicate the amount of information each item provides across the latent trait continuum. Higher information values correspond to lower measurement standard errors and greater measurement precision. The IICs suggest that the items provided information across a range of well-being levels.

**Figure 4:**
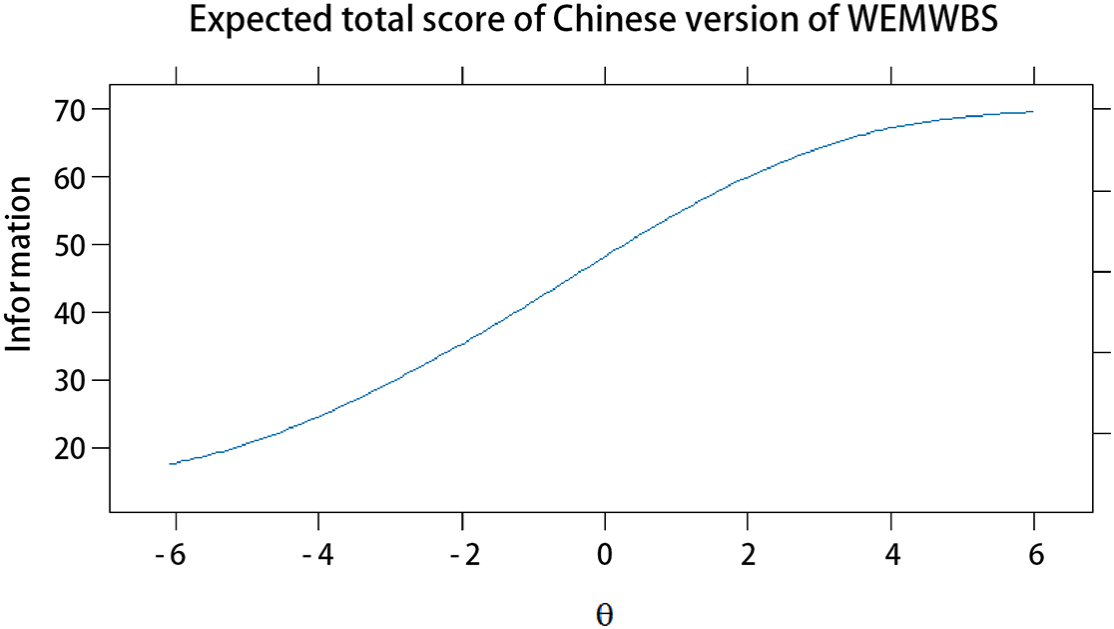
Expected total score curve for the Chinese-language WEMWBS (N = 392).

The expected total score curve in **Figure 4** showed a monotonic increase across latent trait levels, indicating that higher latent well-being corresponded to higher expected WEMWBS total scores.

Overall, the IRT analysis indicated satisfactory discriminatory power for most WEMWBS items. The threshold and information curves provide item-level evidence on how the scale functions across the well-being continuum.

## 4 Discussion

### 4.1 Principal findings

This study evaluated the psychometric properties of the Chinese-language WEMWBS in patients with heart failure or myocardial infarction using an integrated CTT and IRT framework. The findings provide scale-level evidence for reliability and validity and item-level evidence regarding discrimination, thresholds, and information. Together, the results support the potential use of the Chinese-language WEMWBS as a patient-reported measure of positive mental well-being in this cardiovascular clinical population.

Within the CTT framework, the Chinese-language WEMWBS showed excellent internal consistency and criterion-related validity, as indicated by its strong correlation with the WHO-5. EFA supported a dominant one-factor structure, and CFA provided further support for a unidimensional model after theoretically justified correlated residuals were included. Although RMSEA remained above strict conventional thresholds, CFI and SRMR indicated acceptable fit, supporting cautious use of the total score as a composite measure of positive mental well-being.

IRT provided complementary information about the relationship between individual items and the latent trait of mental well-being (22, 40). Under the GRM, most items showed satisfactory discrimination and ordered thresholds, suggesting that response categories functioned as expected. Item information findings further suggested that the scale provides useful measurement information across relevant levels of the well-being continuum.

These findings have potential implications for cardiovascular care. Patients with heart failure or myocardial infarction often experience persistent symptoms, recurrent healthcare use, and psychosocial adjustment challenges (41). The Chinese-language WEMWBS may provide a brief tool for assessing positive mental well-being during routine care, nursing follow-up, and cardiac rehabilitation. Because the WEMWBS assesses positive mental well-being rather than anxiety or depressive symptoms, it should be positioned as a complementary instrument to symptom-focused measures rather than as a substitute. Future research should evaluate its responsiveness, longitudinal stability, and predictive validity before stronger claims about clinical utility are made.

### 4.2 Item discrimination and ceiling effects

The IRT analysis showed item discrimination parameters ranging from 1.29 to 3.83, indicating moderate to high discrimination for most items. Items 8 (a = 3.83), 14 (a = 3.56), and 10 (a = 3.28) showed the highest discrimination, whereas item 4 (a = 1.29) showed the lowest discrimination. The relatively lower discrimination of item 4 (“I’ve been feeling interested in other people”) may reflect contextual constraints in hospitalized cardiovascular patients, whose social opportunities may be limited by illness and treatment. Responses to this item may therefore reflect the immediate care environment as well as underlying mental well-being.

Threshold parameters ranged from –2.61 to 2.77 and increased monotonically across response categories, consistent with the GRM. Items 9 (“I’ve been feeling close to other people”) and 12 (“I’ve been feeling loved”) showed response patterns suggesting higher endorsement of interpersonal connectedness items. This may reflect social desirability, increased family or healthcare support during hospitalization, or cultural expectations around interpersonal closeness. These explanations remain speculative and should be examined in future qualitative and psychometric studies.

Regarding distributional properties, no floor effects were observed, whereas several items showed ceiling effects greater than 15%. Ceiling effects suggest that the scale may have reduced precision for differentiating among patients with higher levels of positive mental well-being. This limitation is relevant for longitudinal follow-up and intervention studies, where ceiling effects may reduce sensitivity to detect further improvements in mental well-being.

In summary, although the Chinese-language WEMWBS demonstrated satisfactory overall psychometric performance in this population, the relatively low discrimination of item 4 and the ceiling effects observed in several items highlight potential measurement limitations that warrant further study.

Future research should further validate the measurement performance of items 4, 9, and 12 in independent cardiovascular samples, consider whether contextual factors influence responses to socially oriented items, supplement self-report data with qualitative interviews or behavioral observations, and examine responsiveness to clinical change in intervention studies.

### 4.3 Comparisons with previous studies

The original WEMWBS was developed to assess positive mental well-being, including positive affect, psychological functioning, and interpersonal relationships (12). Previous studies in different countries and populations have generally supported its reliability and validity (42, 43). The present study extends this evidence to Chinese-speaking patients with heart failure or myocardial infarction and provides support for its potential use in cardiovascular clinical populations.

A unidimensional structure has been supported in the original validation study and in several international studies. However, findings for the Chinese-language WEMWBS have not been entirely consistent, possibly because of differences in culture, translation, interpretation, sample characteristics, and statistical methods. In the present study, EFA and CFA supported a one-factor structure, suggesting that the total score can represent overall positive mental well-being in this population, although the modified CFA model should be replicated in independent samples.

Prior Chinese studies have evaluated the WEMWBS in older adults, university students, medical staff, and patients with chronic heart failure (44). These studies generally reported good internal consistency and validity. However, evidence in broader cardiovascular populations remains limited, especially among patients with myocardial infarction. Patients with heart failure or myocardial infarction may experience long-term symptoms, functional limitations, repeated healthcare use, prognostic uncertainty, and psychological burden, which may influence responses to well-being items. This supports the need for validation in this specific clinical population.

Our findings are generally aligned with prior Chinese studies. Cronbach’s α was high, and the correlation between the Chinese-language WEMWBS and the WHO-5 was strong, supporting internal consistency and criterion-related validity. This study further extends previous research by combining CTT and IRT in patients with heart failure or myocardial infarction. CTT provided evidence on overall scale performance, whereas IRT provided item-level evidence on discrimination, thresholds, and information.

### 4.4 Implications for cardiovascular healthcare

The Chinese-language WEMWBS may be incorporated into cardiovascular nursing assessment, discharge planning, cardiac rehabilitation, and outpatient follow-up to assess positive mental well-being. As a complement to symptom-based measures, it may support more comprehensive, patient-centered care.

The WEMWBS differs from traditional symptom-focused instruments. Measures such as the HADS, PHQ-9, and GAD-7 primarily assess anxiety and depressive symptoms, whereas the WEMWBS focuses on positive mental well-being. This distinction is important because cardiovascular care should not only identify psychological distress but also assess positive psychological resources, including emotional well-being, psychological functioning, and social connectedness (45). Therefore, the WEMWBS may complement anxiety and depression scales in cardiovascular care.

In practical terms, the Chinese-language WEMWBS may help healthcare providers identify patients with lower positive well-being, guide psychosocial support, and evaluate changes in positive mental health during rehabilitation and long-term disease management. It may also serve as a patient-reported outcome measure in studies of psychosocial or behavioral interventions in cardiovascular settings, although responsiveness and predictive validity require further evaluation.

### 4.5 Limitations and future research

This study has several limitations. First, the cross-sectional design precluded evaluation of test–retest reliability, responsiveness, and predictive validity. Future longitudinal studies should examine whether changes in Chinese-language WEMWBS scores reflect changes in psychological well-being during cardiac rehabilitation, nursing follow-up, and psychosocial interventions.

Second, the sample was limited to patients with heart failure or myocardial infarction recruited from tertiary hospitals. Patients treated in tertiary hospitals may have more complex or severe conditions than those treated in community or secondary hospitals, which may affect generalizability. Differences in socioeconomic status, health literacy, and healthcare access may also limit the applicability of findings to broader clinical populations. In addition, voluntary participation may have introduced healthy volunteer bias if individuals with better psychological well-being or greater health awareness were more likely to participate.

Third, although this study integrated CTT and IRT, item-level findings require replication. Items 9 and 12 were endorsed more positively, suggesting distinct response patterns for social connectedness items in cardiovascular patients. Item revision should be approached cautiously given the scale’s established status. Future work should examine ICCs, IICs, local dependence, and DIF in independent samples. The modified CFA model included four pairs of correlated residuals; although these were conceptually justified and supported by modification indices, they remain a limitation that should be addressed through cross-validation without such adjustments.

Fourth, excluding participants with diagnosed severe psychiatric disorders may limit generalizability to patients with heart failure or myocardial infarction and severe psychiatric comorbidities. However, patients with mild-to-moderate depressive or anxiety symptoms were not excluded. Future studies should examine the factor structure of the WEMWBS in cardiovascular patients across varying levels of psychological symptom severity.

Finally, external validation was limited to the WHO-5. Future studies should incorporate anxiety, depression, quality of life, social support, and rehabilitation outcomes to broaden validation evidence and assess clinical utility. Intervention studies are also needed to evaluate the WEMWBS as a patient-reported outcome for psychosocial and behavioral interventions in cardiovascular populations.

## 5 Conclusions

The Chinese-language WEMWBS demonstrated satisfactory psychometric properties in patients with heart failure or myocardial infarction. These findings support its potential use as a patient-reported outcome measure for assessing positive mental well-being in this clinical population. Because the WEMWBS captures positive psychological resources rather than psychological distress, it should be regarded as a complementary tool rather than a replacement for symptom-based measures. Further longitudinal and intervention studies are needed to evaluate its responsiveness, predictive validity, and clinical utility in cardiovascular care.

## Data Availability

The raw data supporting the conclusions of this article will be made available by the authors, without undue reservation.
